# Basophil allergen threshold sensitivity to casein (casein‐specific CD‐sens) predicts allergic reactions at a milk challenge in most but not all patients

**DOI:** 10.1002/iid3.1265

**Published:** 2024-05-09

**Authors:** Solveig Røisgård, Anna Nopp, Anna Lindam, SWITCH study group, Caroline A. Nilsson, Christina E. West

**Affiliations:** ^1^ Department of Clinical Sciences Pediatrics, Umeå University Umeå Sweden; ^2^ Clinical Science and Education Karolinska Institutet Stockholm Sweden; ^3^ Sachs Children and Youth Hospital Södersjukhuset Stockholm Sweden; ^4^ Department of Public Health and Clinical Medicine, Unit of Research, Education and Development Östersund Umeå University Umeå Sweden

**Keywords:** basophil activation, casein, CD‐sens, cow's milk allergy, food challenge

## Abstract

**Background:**

The basophil activation test is an emerging clinical tool in the diagnosis of cow's milk allergy (CMA). The aim was to assess the association between the basophil allergen threshold sensitivity to the major milk protein casein (casein‐specific CD‐sens), the levels of milk‐ and casein‐specific Immunoglobulin E antibodies (IgE‐ab), and the severity of allergic reactions at milk challenges.

**Methods:**

We enrolled 34 patients aged 5–15 (median 9) years who underwent a double‐blind placebo‐controlled milk‐challenge (DBPCMC) as screening before inclusion in an oral immunotherapy study for CMA. The severity of the allergic reaction at the DBPCMC was graded using Sampson's severity score. Venous blood was drawn before the DBPCMC. Milk‐ and casein‐specific IgE‐ab were analyzed. Following in vitro stimulation of basophils with casein, casein‐specific CD‐sens, was determined.

**Results:**

Thirty‐three patients completed the DBPCMC. There were strong correlations between casein‐specific CD‐sens and IgE‐ab to milk (*r*_s_ = 0.682, *p* < .001), and between casein‐specific CD‐sens and IgE‐ab to casein (*r*_s_ = 0.823, *p* < .001). There was a correlation between the severity of the allergic reaction and casein‐specific CD‐sens level (*r*_s_ = 0.395, *p* = .041) and an inverse correlation between casein‐specific CD‐sens level and the cumulative dose of milk protein to which the patient reacted at the DBPCMC (*r*_s_ = −0.418, *p* = .027). Among the 30 patients with an allergic reaction at the DBPCMC, 67% had positive casein‐specific CD‐sens, 23% had negative casein‐specific CD‐sens, and 10% were declared non‐responders.

**Conclusion:**

Two thirds of those reacting at the DBPMC had positive casein‐specific CD‐sens, but reactions also occurred despite negative casein‐specific CD‐sens. The association between casein‐specific CD‐sens and the severity of the allergic reaction and cumulative dose of milk protein, respectively, was moderate.

## Graphical abstract

We enrolled 34 patients aged 5–15 (median 9) years who underwent a DBPCMC as screening before inclusion in an oral immunotherapy study for milk allergy. In summary, patients with a high level of CD‐sens tolerated a smaller amount of milk protein at the DBPCMC (*r* = −0.418, *p*= .027).

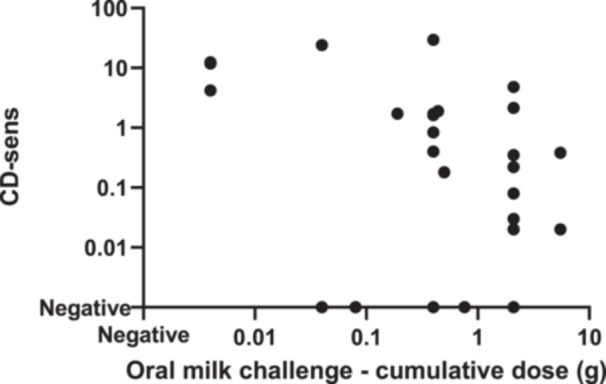

## 1 BACKGROUND

Cow's milk allergy (CMA) affects less than 1% of infants^1^ and can be classified into Immunoglobulin E (IgE)‐mediated, non‐IgE‐mediated and mixed reactions. The immediate allergic reactions are mediated by specific IgE‐antibodies (IgE‐ab) and can potentially trigger anaphylaxis. Although the natural resolution of CMA is high^2^, ^3^ there are children that have a more persistent phenotype requiring repeated clinical evaluation of tolerance development. CMA can be confirmed by an allergic reaction at an oral food challenge (OFC), and this is considered the gold standard.^4^ To categorize IgE‐mediated allergic reactions at an OFC, Sampson's score,^5^ which is a widespread method for grading of anaphylaxis, can be used.

Because the OFC procedure is time consuming, costly and comes with a risk of severe allergic reactions, there is a need of complementary diagnostic tools.^6^ The level of milk specific IgE‐ab and its components are associated to allergic reactions in CMA but can differ widely across individuals and do not predict the severity of the allergic reaction.^4^ Casein, the major component, is potentially the best performing diagnostic component in the diagnosis of CMA but there are no valid biomarkers that sufficiently can predict tolerance or the risk of severe allergic reactions at the OFC.^7^, ^8^ A potential biomarker for clinical use, is basophil activation and in vitro stimulation of basophils with casein, could potentially mirror allergic reactions to milk proteins in vivo better than IgE‐ab to milk and casein.^6^, ^7^, ^8^, ^9^, ^10^, ^11^ Basophil activation can be measured as basophil reactivity,^12^ the number of basophils that respond to a given stimulus, and basophil sensitivity,^13^ the allergen concentration at which half of all reactive basophils respond. Evaluation of basophil allergen sensitivity requires measurement of reactivity at 6–8 allergen concentrations.^14^ Previous studies demonstrate a correlation between basophil activation and allergic reactions to food proteins,^9^, ^10^, ^11^, ^15^, ^16^ but more studies in cow's milk allergic patients are needed to assess the possible advantages of this functional analysis for diagnosis and prediction of allergic reactions to cow's milk and in monitoring desensitization to this food.^17^, ^18^, ^19^

The objective of this explorative and descriptive study was to assess the potential association between casein‐specific CD‐sens, the levels of milk‐ and casein‐specific IgE‐ab, and the severity of allergic reactions at milk challenges.

## 2 METHODS

### 2.1 Study design and population

We enrolled 34 patients who had a convincing clinical history (presenting symptoms ranging from local symptoms to anaphylaxis) and a diagnosis of IgE‐mediated CMA. To ascertain the CMA diagnosis, the patients underwent a double‐blind placebo‐controlled milk‐challenge (DBPCMC) as screening before inclusion in an open randomized controlled trial (RCT) (ClinicalTrials ID NCT03819556) of oral immunotherapy for CMA. Inclusion criteria to participate in this study were: a clinical diagnosis of CMA with IgE‐ab to milk >0.1 kU_A_/L, total elimination of milk products from the diet and age 5–15 years. The main exclusion criteria were uncontrolled asthma, that is, spirometry demonstrating >12% reversibility in forced expiratory volume in the first second (FEV1) and/or asthma control test (ACT) < 20 points, symptoms of an ongoing infection or allergy. Other exclusion criteria were severe immunodeficiency, autoimmune disease, chronic urticaria and eosinophilic esophagitis. Oral antihistamines were not allowed 3 days before the DBPCMC, and oral corticosteroids were not allowed a week before the challenge session. This multicenter trial was conducted at seven sites in Sweden, and subjects were recruited from April 2018 to March 2021. Written informed consent was obtained from parents and from subjects who were 15 years old when entering the study. The research was approved by the Ethical Review Authority (2016‐517‐31M, 2018‐502‐32M and 2021‐06692‐02). The study was conducted according to the Declaration of Helsinki.

### 2.2 Double‐blind placebo‐controlled OFC with milk

The DBPCMC was performed with powdered milk (Semper, a part of Hero Group) and the challenge matrix consisted of an amino acid‐based formula, Elemental 028 (Nutricia, a part of Danone, division for Advanced Medical Nutrition). As a placebo, rice flour (Risenta, a part of the Paulig Group) was added to the challenge matrix. The patients, parents, study nurses, and study doctors were all blinded. Only the dietician preparing the challenge knew if the participant was given an active or placebo substance. The DBPCMC was performed on separate days with a minimum interval of two consecutive days and a maximum interval of 2 weeks. The doses were given in five steps, starting with 0.0036 g milk protein. The maximum dose was 3.4 g (94 mL), and the cumulative dose was 5.5 g (152 mL) of milk protein.^20^ The challenge was stopped when objective allergic symptoms appeared. The symptoms (skin, gastrointestinal (GI), respiratory, cardiovascular, and neurological) were registered in a standardized protocol for symptom registration and categorized according to Sampson's score. Every organ manifestation in Sampson's score is graded from 1 (mild) to 5 (severe), and if multiple symptoms, the grading was related to the organ system mostly affected. The DBPCMC day was considered negative if the planned doses were given with no clinical signs or symptoms and were considered positive when there was an objective allergic reaction to the active substance and no reaction to placebo.

### 2.3 Blood sampling procedures and determination of IgE

Venous blood was drawn before the first DBPCMC. From serum gel CAT tubes, specific IgE‐ab to cow's milk and casein were analyzed using ImmunoCAP (Thermo Fisher Scientific/Phadia) according to the manufacturer's instructions.

### 2.4 Basophil activation test (CD‐sens)

Blood drawn into heparin tubes was sent to the Research Center at Södersjukhuset in Stockholm and analyzed within 24 h. One hundred microliters blood was mixed with 100 µL B casein extract (final concentration 0.25–25,000 ng/mL) (CAS 9000‐71‐9, Sigma‐Aldrich), anti‐IgE antibody 1 µg/mL (positive control) (Beckman Coulter, Inc.) or RPMI (negative control) (cell culture media developed at Roswell Park Memorial Institute) and incubated in water bath +37°C for 20 min. The stimulation was terminated by placing the tubes on ice for 5 min followed by incubated 25 min on ice in the dark with CD203c‐PE (identification marker) (Immunotech) and CD63‐FITC (activation marker) (Immunotech). After antibody staining, the red blood cells were haemolysed by adding 2 mL of +4°C isotonic NH4Cl‐EDTA lyzing solution (154 mM NH4Cl, 10 mM KHCO_3_, 0.1 mM EDTA, pH 7.2). The leukocyte suspensions were then centrifuged at 300 × *g* for 5 min at +4°C and washed with 2 mL of +4°C 0.15 M phosphate‐buffered saline (PBS), pH 7.4, supplemented with 0.02% NaN3. After centrifugation, the supernatant was removed, and the leukocyte pellet was re‐suspended in 300 μL PBS and analyzed in a flow cytometer. A minimum of 200 basophils/tube was analyzed.

**FIGURE 1:**
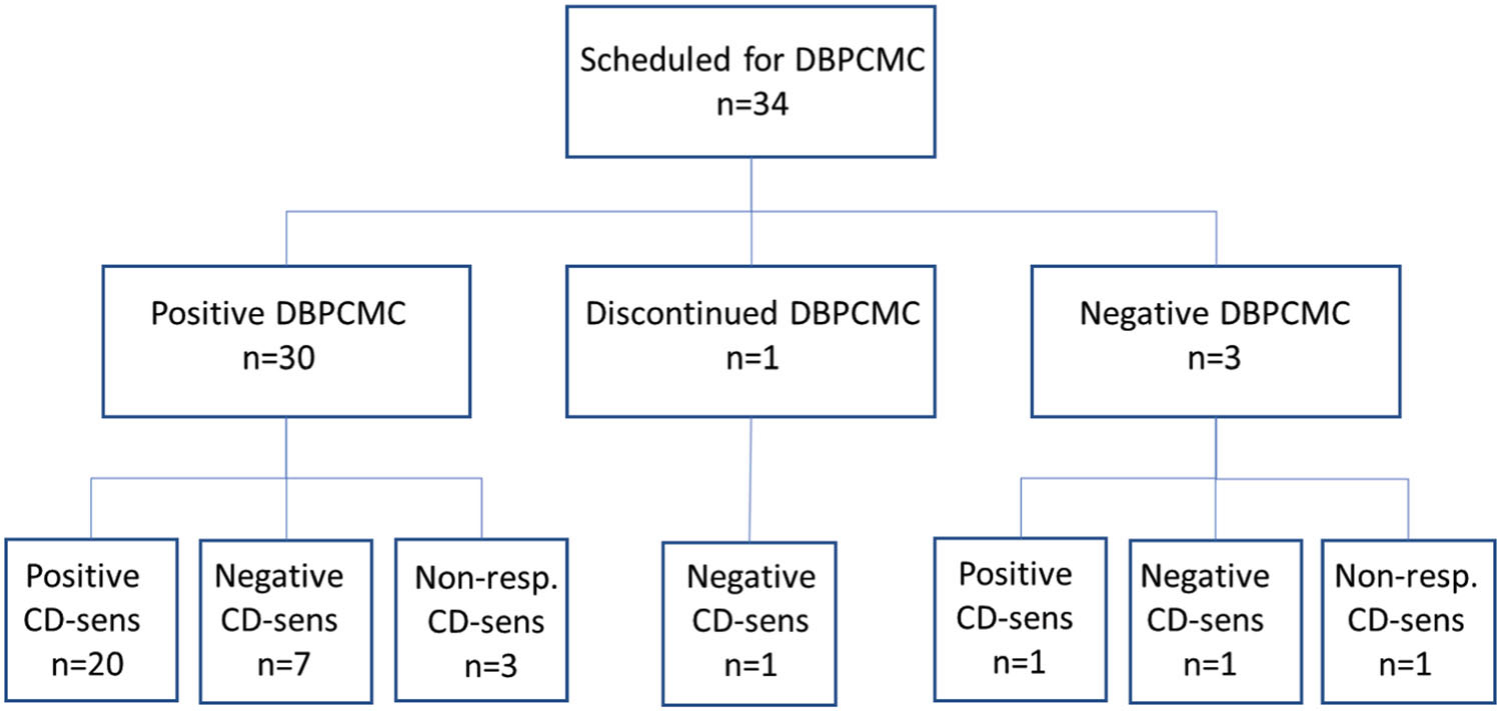
Flow chart of the study participants.

**FIGURE 2:**
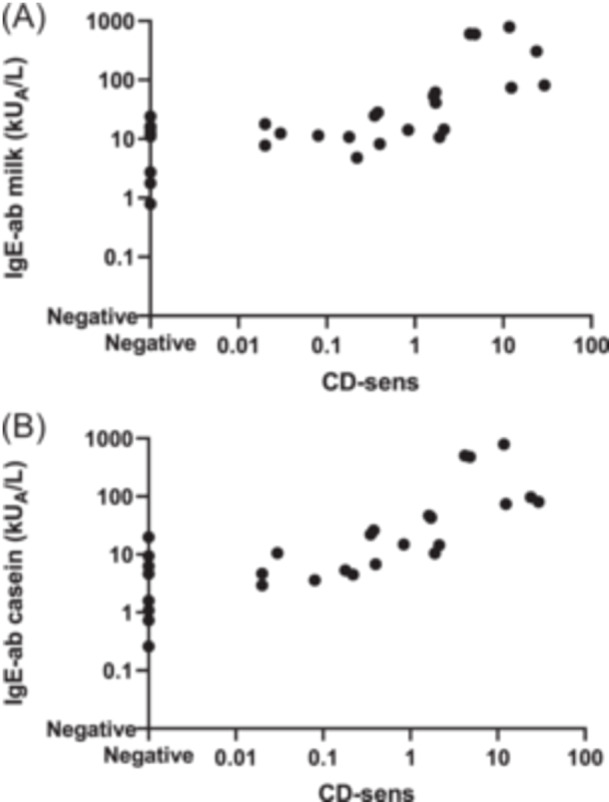
There were strong positive correlations between (A) basophil sensitivity (casein‐specific CD‐sens) and IgE‐antibodies (IgE‐ab) to milk (*r*_s_ = 0.682, *p* < .001), and between (B) casein‐specific CD‐sens and IgE‐ab to casein (*r*_s_ = 0.823, *p* < .001).

The cut‐off determining a positive test was set to 10% of CD63‐positive basophils. Patients, whose basophils after stimulation with the positive control (anti‐IgE) responded with less than 10% CD63 upregulation, were regarded as non‐responders. To determine the basophil allergen threshold sensitivity, CD‐sens, the eliciting allergen concentration giving 50% (EC50) of maximum CD63% upregulation of the dose–response curve is calculated (see Figures 1, 2). CD‐sens definition: the inverted value for EC50 multiplied by 100.^21^

### 2.5 Statistical analysis

As the outcome variables were ordinal or not normally distributed, statistical analyses were performed using non‐parametric tests. Descriptive statistics are presented with medians, interquartile range, range, or percent. Spearman's rank order correlation was used to analyze the potential correlation between casein‐specific CD‐sens, IgE‐ab and Sampson's score and between casein‐specific CD‐sens, IgE‐ab, and cumulative milk dose. A significance level of 0.05 was assumed, and the Statistical software SPSS version 28.0.1.0 was used for all analyses. Statistical power was calculated a priori for the main outcome of the RCT based on the results in the Cochrane analysis for OIT against milk allergy.^22^ To be able to demonstrate a difference between a tolerance level of 10% for the control group and 40% for the active group (80% power, 5% significance level) 32 study participants would be needed in the respective group. No statistical power calculation was made for this explorative and descriptive study. The figures were created in GraphPad Prism 8.3 (GraphPad Software, Inc.).

## 3 RESULTS

### 3.1 Patients and outcome of the DBPCMC

Among the included 34 patients, 59% were boys and in median of 9 years old (range 5–15 years). The patient characteristics are displayed in Table 1. Of the 34 patients who underwent the DBPCMC, 30 had an allergic reaction, while three did not react and were declared tolerant. One patient discontinued the DBPCMC (Figure 1). The IgE‐ab levels to milk in patients who were tolerant to milk at the DBPCMC were 28.1, 10.8, and 0.8 kU_A_/L to milk, respectively. The corresponding numbers of IgE‐ab levels to casein were 26.0, 10.5, and 0.5 kU_A_/L. There were no reactions to placebo.

**TABLE 1:** Characteristics of the study patients.

Characteristics	Total; *n* = 34
Age; y, median (range)	9 (5–15)
Sex; m, *n* (%)	20 (59)
IgE‐ab milk; kU_A_/L, median (range)	13.4 (0.8–793)
IgE‐ab casein; kU_A_/L, median (range)	8.1 (0.3–793)
CD‐sens casein; median (range)	4.7 (0.02–24)
Asthma; *n* (%)	28 (82)
Eczema; *n* (%)	13 (38)
Other food allergy; *n* (%)	28 (82)
Pollen allergy; *n* (%)	23 (68)
Fur animal allergy; *n* (%)	17 (50)
Milk consumption before DBPCMC; *n* (%)	0 (0)
Positive DBPCMC; *n* (%)	30 (88)
Cumulative milk dose; mL, median (range)	11.1 (0.1–152)
Negative DBPCMC; *n* (%)	3 (9)

Abbreviations: DBPCMC, double‐blind placebo‐controlled milk‐challenge; IgE‐ab, Immunoglobulin E antibody.

Of the 30 patients with an allergic reaction at the DBPCMC, 25 (83%) had oro‐laryngeal symptoms, 23 (77%) had skin manifestations (localized pruritus, flushing, urticaria), and 18 (60%) had gastrointestinal symptoms (abdominal pain, nausea, vomiting). Other manifestations were less frequent: 10 patients (33%) had rhinoconjunctivitis, 9 patients (30%) had respiratory, and 4 patients (13%) had circulatory manifestations. The reactions were classified by Sampson's severity score: grade 1, 7 patients (23%); grade 2, 10 patients (33%); grade 3, 12 patients (40%); and grade 5, 1 patient.

### 3.2 Casein‐specific CD‐sens, IgE‐ab to milk and casein, and the prediction of an allergic reaction at the DBPCMC

For all 33 patients who completed the DBPCMC, casein‐specific CD‐sens was analyzed; of these, 21 (64%) were positive in casein‐specific CD‐sens, 8 (24%) were negative, and 4 (12%) were classified as non‐responders and declared as missing in the analyses (Table 2). Among the 30 patients reacting with an allergic reaction at the DBPCMC, the majority (67%) had a positive casein‐specific CD‐sens, but 7 (23%) had an allergic reaction (grade 1–3 according to Sampson's severity score and with a cumulative milk dose ranging from 1.1 to 58.1 mL) at the DBPCMC despite a negative casein‐specific CD‐sens. Three patients (10%) of those reacting at the DBPCMC were declared non‐responders in the casein‐specific CD‐sens analyses. Of the three patients that did not have an allergic reaction at the DBPCMC, one was positive, one was negative, and one was declared as a nonresponder in the casein‐specific CD‐sens analysis (Figure 1).

**TABLE 2:** Challenge outcomes and biomarkers in the study patients (*n* = 33).

DBPCMC	Sampson's score	Cumulative dose (mL)	IgE‐ab milk kU_A_/L	IgE‐ab casein kU_A_/L	IgE kU_A_/L	Casein‐specific CD‐sens
Negative		152	28.1	26	1245	0.38
Negative		152	10.8	4.36	5000	Negative
Negative		152	0.8	0.5	1756	Nonresponder
Positive	3	13.1	10.7	5.31	182	0.18
Positive	3	152	17.8	4.66	492	0.02
Positive	3	0.1	793	793	5000	11.8
Positive	2	58.1	7.75	2.93	1642	0.02
Positive	2	58.1	11.4	3.6	816	0.08
Positive	3	58.1	14.4	14.5	115	2.14
Positive	3	11.1	14.2	14.9	544	0.84
Positive	2	58.1	15.4	4.63	1662	Negative
Positive	3	1.1	306	96.5	775	24
Positive	5	58.1	597	480	1584	4.8
Positive	1	11.1	1.77	0.73	395	Neg
Positive	1	1.1	0.79	0.26	128	Neg
Positive	1	58.1	1.1	1.08	731	Neg
Positive	2	2.1	41	42.7	282	1.72
Positive	2	0.7	12.4	10.5	1472	0.03
Positive	2	58.1	24.1	19.8	618	Negative
Positive	1	21.1	6.75	0.62	455	Nonresponder
Positive	3	12.1	10.7	10.4	73	1.9
Positive	3	58.1	16.3	9.38	1720	Negative
Positive	1	47	4.78	4.47	1009	0.22
Positive	1	2.7	2.77	1.8	174	Nonresponder
Positive	1	47	24.7	22.1	1034	0.35
Positive	2	11.1	53.6	46.6	290	1.63
Positive	3	11.1	61.8	44.3	182	1.71
Positive	3	11.1	81	80.8	321	29.3
Positive	1	0.1	74	74	2559	12.4
Positive	2	6.1	12.5	0.41	151	Nonresponder
Positive	2	0.1	603	504	1929	4.21
Positive	3	11.2	8.25	6.81	152	0.4
Positive	3	11.1	2.72	1.6	718	Negative

Abbreviations: DBPCMC, double‐blind placebo‐controlled milk‐challenge; IgE‐ab, immunoglobulin E antibody.

We analyzed the correlation between casein‐specific CD‐sens, and IgE‐ab to milk and casein, respectively. There was a strong positive correlation between casein‐specific CD‐sens and IgE‐ab to milk (*r*_s_ = 0.682, *p* < .001), and an even stronger correlation between casein‐specific CD‐sens and IgE‐ab to casein (*r*_s_ = 0.823, *p* < .001) (Figure 2A,B).

**FIGURE 3:**
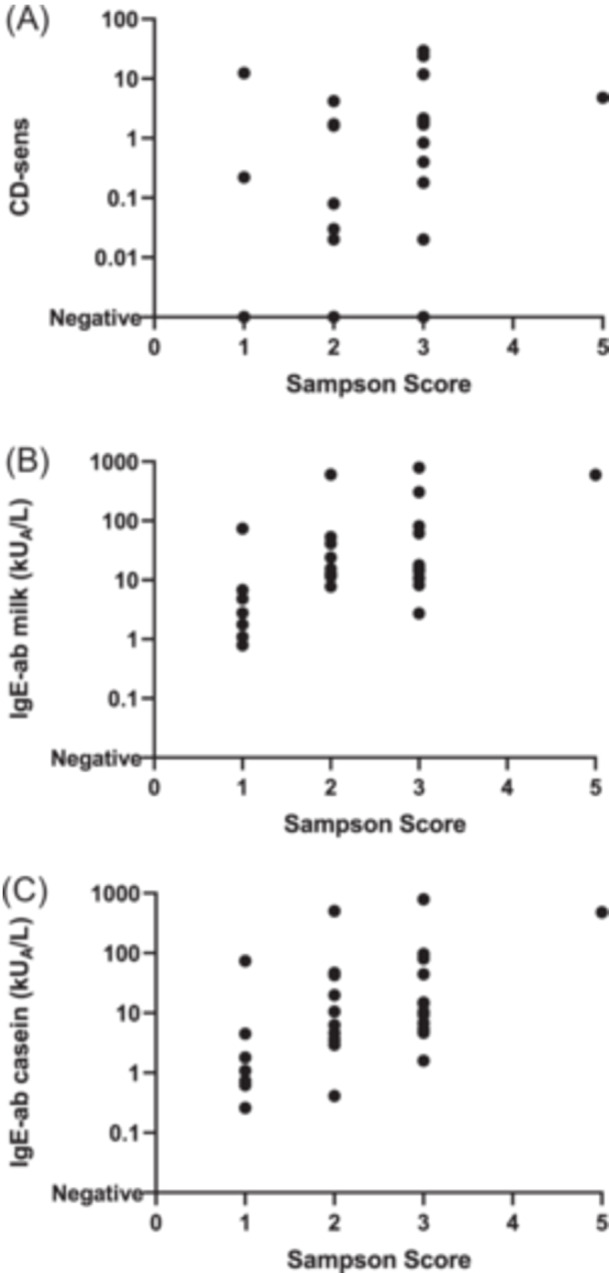
There was a moderate positive correlation between the severity of the allergic reaction (assessed by Sampson's severity score) and (A) basophil sensitivity (casein‐specific CD‐sens) (*r*_s_ = 0.395, *p* = .041), (B) IgE‐antibodies (IgE‐ab) to milk (*r*_s_ = 0.442, *p* = .015) and (C) IgE‐ab to casein (*r*_s_ = 0.516, *p* = .004).

**FIGURE 4:**
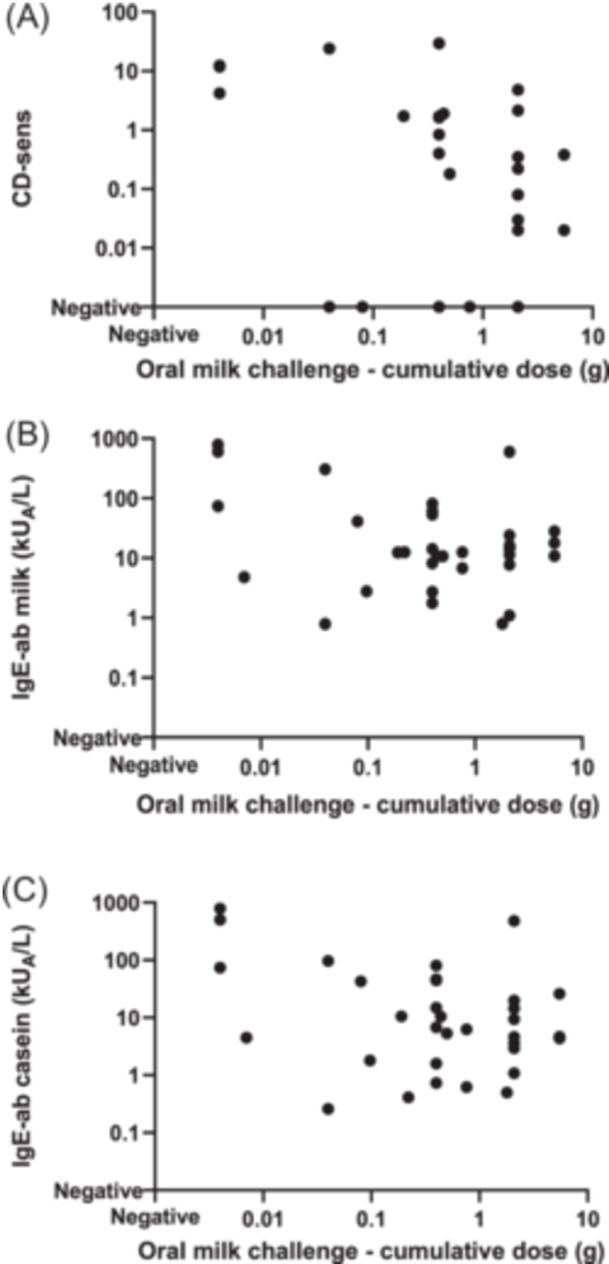
A lower cumulative milk dose correlated inversely to (A) higher basophil sensitivity (casein‐specific CD‐sens) (*r*_s_ = −0.418, *p* = .027) but not to (B) IgE‐ab to milk (*r*_s_  = −0.145, *p* = .429) or (C) IgE‐ab to casein, respectively (*r*_s_ = −0.265, *p* = .143).

Next, we analyzed the correlation between casein‐specific CD‐sens and the severity of allergic symptoms at the DBPCMC according to Sampson's severity score.^5^ We saw a moderate positive correlation between the severity of the allergic reaction and the casein‐specific CD‐sens level (*r*_s_ = 0.395, *p* = .041). The higher the casein‐specific CD‐sens, the more severe the symptoms graded by Sampson (Figure 3A). There was also a positive correlation between the severity of allergic reactions, by Sampson's severity score, and the level of IgE‐ab to milk (*r*_s_ = 0.442, *p* = .015) but especially between IgE‐ab to casein and Sampson's severity score (*r*_s_ = 0.516, *p* = .004) (Figure 3B,C). There was a moderate inverse correlation between casein‐specific CD‐sens and the cumulative dose of milk protein to which the patient reacted at the DBPCMC (*r*_s_ = −0.418, *p* = .027) (Figure 4A). Thus, patients with a high level of casein‐specific CD‐sens, that is, the basophils reacting at a lower casein concentration, tolerated a smaller amount of milk protein at the DBPCMC. Although the difference was not statistically significant, a similar pattern could be seen when we analyzed the correlation between cumulative milk dose and IgE‐ab to milk (*r*_s_ = −0.145, *p* = .429) and IgE‐ab to casein, respectively (*r*_s_ =−0.265, *p* = .143) (Figure 4B,C).

## 4 DISCUSSION

Today there is no in vitro diagnostic test for CMA that captures the broad spectrum of allergic symptoms that may arise, ranging from local symptoms to anaphylaxis. For allergens from the plant kingdom, that is, peanuts, the introduction of analyses of IgE‐ab against individual proteins (components) has revolutionized the diagnostics with both high sensitivity and specificity.^23^, ^24^ Similar concepts have been tested for milk, egg, and other animal allergens, but since these allergens have a more complex sensitization pattern and the proteins have a more complex structure, well‐functioning components with high sensitivity and specificity have not yet been produced.^8^ Consequently, this has led to the development of other diagnostic tests which are based on analysis of basophils. In this explorative study, we investigated the potential advantages of casein‐specific CD‐sens in predicting the severity of the allergic reaction during a milk challenge.

In vitro stimulation of basophils with casein, could potentially mirror allergic reactions to milk proteins in vivo better than IgE‐ab to milk and casein.^6^, ^7^, ^8^, ^9^, ^10^, ^11^ Indeed, we found that patients with a high level of casein‐specific CD‐sens, that is, the basophils reacting at a lower casein concentration, tolerated a smaller amount of milk protein at the DBPCMC. A similar pattern was seen for IgE‐ab to milk and casein, but here, the inverse correlations were weaker and not statistically significant. Thus, our findings suggest that casein‐specific CD‐sens can predict if the patient is likely to react at a lower dose of milk protein, which could be of relevance in the decision if a milk challenge should be conducted.

We found that among the 30 patients reacting at the DBPCMC, 67% had a positive casein‐specific CD‐sens, 23% had a negative casein‐specific CD‐sens, and 10% were declared as non‐responders in casein‐specific CD‐sens. The latter is in line with previous studies that report basophil inactivation (“basophil anergy”) in around 10% of patients.^25^ It should be noted that the oral challenges in our study were performed with a whole milk extract containing multiple milk allergens, whereas casein‐specific CD‐sens was performed with casein. The rationale of using casein was to have control of the amount of protein used in the test. Casein is also a stable protein causing symptoms in many milk‐allergic individuals.^26^ However, by using a single allergen, we miss the complexity that exists with whole milk extract, where many different allergens may act together, activating mast cells or basophils, hence resulting in an allergic response. Low levels of several allergens could potentially activate mast cells more easily than a high concentration of a single allergen.^10^, ^11^ Thus, future studies using whole milk extract could give additional information.

To date, most studies have evaluated basophil activation tests (BAT) in peanut allergy.^27^, ^28^ In these studies, specific components as well as the whole peanut extract, have been used. Notably, Ara h 2 reported lower sensitivity in BAT than in the complete peanut extract.^23^ Studies using BAT in IgE‐mediated CMA are very few, and all have used whole milk extract, with a reported sensitivity and specificity of 100% in infants and an area under curve (AUC) of 0.924 in children.^10^, ^11^ However, since one of the inclusion criteria in the present study was a positive oral milk challenge before the start of milk oral immunotherapy, it was not possible to analyze sensitivity/specificity or AUC since negative challenges would be needed for that calculation.

As expected, we found an excellent correlation between casein‐specific CD‐sens and casein‐specific‐IgE‐ab (*r*_s_ = 0.823, *p* < .001). There was also a correlation between casein‐specific CD‐sens and milk‐specific‐IgE‐ab (*r*_s_ = 0.682, *p* < .001). However, this correlation was weaker indicating a more complex pattern of antibodies detected with the whole milk extract in the IgE‐ab analysis as compared to the pure protein casein.

Our study has several strengths, including the prospective design, the DBPCMC, the use of severity scoring and the technical calibration of the CD‐sens analysis. There are possible limitations when it comes to the performance of the challenges and assessment of objective allergic reactions. The severity of the allergic reaction, here graded according to Sampson's score,^5^ will, to a certain extent, be dependent on the physician's tendency to stop the challenge when mild symptoms appear or continue to the next step. Further, the nature of the allergen included in basophil activation will also influence the outcome. We used casein, and it remains undecided how whole milk extract would have influenced the result of CD‐sens compared to casein in this study. In addition, there is an ongoing discussion on how BAT results should be analyzed and interpreted. In this study, we analyzed CD‐sens, but to allow the comparisons of BAT results between laboratories, there is a need of standardization. Finally, the study sample was small.

In summary, patients with a high level of casein‐specific CD‐sens tolerated a smaller amount of milk protein at the DBPCMC. This stands in contrast to milk IgE‐ab and casein IgE‐ab, which could not predict the amount of milk protein tolerated. The symptom severity at the DBPCMC could be predicted by all three biomarkers. A positive casein‐specific CD‐sens result could predict an allergic reaction at the DBPCMC in two thirds of the patients, but allergic reactions also occurred despite negative casein‐specific CD‐sens. Future studies should assess if CD‐sens with whole milk extract performs better than casein‐specific CD‐sens in the risk prediction of allergic reactions at milk challenges. Although using BAT in clinical practice is promising, there is still a need of improved diagnostic tools for the prediction of the severity of allergic reactions at milk challenges and standardization of BAT.

## SWITCH STUDY GROUP

Anders Berg, Department of Pediatrics, Falu Hospital, Falun, Sweden; Mareike Fech‐Bormann, Department of Pediatrics, Västmanland Hospital, Västerås, Stockholm; Emma Goksör, Department of Clinical Science, Pediatrics, Gothenburg University, Gothenburg, Sweden; Daiva Helander, Department of Pediatric Allergy and Pulmonology, Astrid Lindgren's Children's Hospital, Stockholm, Sweden; Lennart Nilsson, Department of Clinical and Experimental Medicin, Center of Allergy, Linköping University, Linköping, Sweden; Carina Uhl, Sachs Children and Youth Hospital, Södersjukhuset, Stockholm, Sweden; Anna Zingmark Terning, Department of Pediatrics, Länssjukhuset Ryhov, Jönköping, Sweden.

## AUTHOR CONTRIBUTIONS

**Solveig Røisgård**: Conceptualization (equal); formal analysis (equal); funding acquisition (equal); investigation (equal); methodology (equal); software (equal); writing—original draft (equal); writing—review and editing (equal). **Anna Nopp**: Data curation (equal); formal analysis (equal); funding acquisition (equal); investigation (equal); methodology (equal); resources (equal); software (equal); writing—original draft (equal); review and editing (equal). **Anna Lindam**: Formal analysis (equal); investigation (equal); methodology (equal); software (equal); validation (equal); writing—review and editing (equal). **Caroline A Nilsson**: Conceptualization (equal); investigation (equal); methodology (equal); resources (equal); writing—review and editing (equal). **Christina E. West**: Conceptualization (equal); data curation (equal); formal analysis (equal); funding acquisition (lead); investigation (equal); methodology (equal); project administration (lead); resources (equal); software (equal); supervision (lead); validation (lead); writing—original draft (equal); writing—review and editing (equal).

## CONFLICT OF INTEREST STATEMENT

CEW has received research funding from Thermo Fisher Scientific, which was directly paid to the institution, and speaker honorarium from Aimmune Therapeutics, a Nestlé Health Science company, outside of the submitted work. CN has received funding from the institution for participating in studies and Advisory board at Aimmune Therapeutics, a Nestlé Health Science company and speaker honorarium from MEDA, ALK, and GSK outside of the submitted work. The other authors declare no conflict of interest.

## ETHICS STATEMENT

The research was approved by the Ethical Review Authority (2016‐517‐31M, 2018‐502‐32M, 2021‐06692‐02).

## CLINICAL TRIAL REGISTRATION

ClinicalTrials ID NCT03819556.

## Supporting information

Supporting information.

## Data Availability

The data that support the findings of this study are available from the corresponding author upon reasonable request.

## References

[1] Schoemaker AA , Sprikkelman AB , Grimshaw KE , et al. Incidence and natural history of challenge‐proven cow's milk allergy in European children—EuroPrevall birth cohort. Allergy. 2015;70(8):963‐972. 25864712 10.1111/all.12630

[2] Elizur A , Rajuan N , Goldberg MR , Leshno M , Cohen A , Katz Y . Natural course and risk factors for persistence of IgE‐mediated cow's milk allergy. J Pediatr. 2012;161(3):482‐7. e1. 22480700 10.1016/j.jpeds.2012.02.028

[3] Winberg A , West CE , Strinnholm Å , Nordström L , Hedman L , Rönmark E . Assessment of allergy to milk, egg, cod, and wheat in Swedish schoolchildren: a population based cohort study. PLoS One. 2015;10(7):e0131804. 26134827 10.1371/journal.pone.0131804 PMC4489866

[4] Muraro A , Werfel T , Hoffmann‐Sommergruber K , et al. EAACI food allergy and anaphylaxis guidelines: diagnosis and management of food allergy. Allergy. 2014;69(8):1008‐1025. 24909706 10.1111/all.12429

[5] Sampson HA . Anaphylaxis and emergency treatment. Pediatrics. 2003;111:1601‐1608. 12777599

[6] Ansotegui IJ , Melioli G , Canonica GW , et al. IgE allergy diagnostics and other relevant tests in allergy, a World Allergy Organization position paper. World Allergy Organ J. 2020;13(2):100080. 32128023 10.1016/j.waojou.2019.100080 PMC7044795

[7] Foong RX , Dantzer JA , Wood RA , Santos AF . Improving diagnostic accuracy in food allergy. J Allergy Clin Immunol Pract. 2021;9(1):71‐80. 33429723 10.1016/j.jaip.2020.09.037 PMC7794657

[8] Foong RX , Santos AF . Biomarkers of diagnosis and resolution of food allergy. Pediatr Allergy Immunol. 2021;32(2):223‐233. 33020989 10.1111/pai.13389

[9] Rubio A , Vivinus‐Nébot M , Bourrier T , Saggio B , Albertini M , Bernard A . Benefit of the basophil activation test in deciding when to reintroduce cow's milk in allergic children. Allergy. 2011;66(1):92‐100. 20608919 10.1111/j.1398-9995.2010.02432.x

[10] Ruinemans‐Koerts J , Schmidt‐Hieltjes Y , Jansen A , Savelkoul HFJ , Plaisier A , van Setten P . The basophil activation test reduces the need for a food challenge test in children suspected of IgE‐mediated cow's milk allergy. Clin Exp Allergy. 2019;49(3):350‐356. 30408255 10.1111/cea.13307

[11] Kim YH , Kim YS , Park Y , et al. Investigation of basophil activation test for diagnosing milk and egg allergy in younger children. J Clin Med. 2020;9(12):3942. 33291359 10.3390/jcm9123942 PMC7762017

[12] Sainte‐Laudy J , Vallon C , Guérin JC . Analysis of membrane expression of the CD63 human basophil activation marker. Applications to allergologic diagnosis. Allerg Immunol. 1994;26(6):211‐214. 7524523

[13] Johansson SGO , Nopp A , van Hage M , et al. Passive IgE‐sensitization by blood transfusion. Allergy. 2005;60(9):1192‐1199. 16076307 10.1111/j.1398-9995.2005.00870.x

[14] Schmid JM , Würtzen PA , Dahl R , Hoffmann HJ . Early improvement in basophil sensitivity predicts symptom relief with grass pollen immunotherapy. J Allergy Clin Immunol. 2014;134(3):741‐4. e5. 24934275 10.1016/j.jaci.2014.04.029

[15] Glaumann S , Nopp A , Johansson SGO , Rudengren M , Borres MP , Nilsson C . Basophil allergen threshold sensitivity, CD‐sens, IgE‐sensitization and DBPCFC in peanut‐sensitized children. Allergy. 2012;67(2):242‐247. 22126416 10.1111/j.1398-9995.2011.02754.x

[16] Song Y , Wang J , Leung N , et al. Correlations between basophil activation, allergen‐specific IgE with outcome and severity of oral food challenges. Ann Allergy Asthma Immunol. 2015;114(4):319‐326. 25841330 10.1016/j.anai.2015.01.006

[17] Santos AF , Shreffler WG . Road map for the clinical application of the basophil activation test in food allergy. Clin Exp Allergy. 2017;47(9):1115‐1124. 28618090 10.1111/cea.12964 PMC5601249

[18] Nucera E , Pecora V , Buonomo A , et al. Utility of basophil activation test for monitoring the acquisition of clinical tolerance after oral desensitization to cow's milk: pilot study. United European Gastroenterol J. 2015;3(3):272‐276. 10.1177/2050640615570694 PMC4480537 26137302

[19] Chirumbolo S . Basophil activation test in oral desensitization to cow's milk allergy. United European Gastroenterol J. 2016;4(5):714‐715. 10.1177/2050640615614793 PMC5042305 27733914

[20] Society SP Komjölksprovokation 2017. Retrieved December 1, 2023, from https://aol.barnlakarforeningen.se/wp-content/uploads/sites/24/2020/07/C6_km_provokation.pdf

[21] Nopp A , Johansson SGO , Ankerst J , et al. Basophil allergen threshold sensitivity: a useful approach to anti‐IgE treatment efficacy evaluation. Allergy. 2006;61(3):298‐302. 16436137 10.1111/j.1398-9995.2006.00987.x

[22] Yeung JP , Kloda LA , McDevitt J , Ben‐Shoshan M , Alizadehfar R . Oral immunotherapy for milk allergy. Cochrane Database Syst Rev. 2012;11(11):CD009542. 23152278 10.1002/14651858.CD009542.pub2 PMC7390504

[23] Chapuis A , Thevenot J , Coutant F , et al. Ara h 2 basophil activation test does not predict clinical reactivity to peanut. J Allergy Clin Immunol Pract. 2018;6(5):1772‐4. e1. 29410305 10.1016/j.jaip.2018.01.021

[24] Petersen TH , Mortz CG , Bindslev‐Jensen C , Eller E . Cow's milk allergic children—can component‐resolved diagnostics predict duration and severity? Pediatr Allergy Immunol. 2018;29(2):194‐199. 29314279 10.1111/pai.12854

[25] Puan KJ , Andiappan AK , Lee B , et al. Systematic characterization of basophil anergy. Allergy. 2017;72(3):373‐384. 27271846 10.1111/all.12952

[26] Cuomo B , Indirli GC , Bianchi A , et al. Specific IgE and skin prick tests to diagnose allergy to fresh and baked cow's milk according to age: a systematic review. Ital J Pediatr. 2017;43(1):93. 29025431 10.1186/s13052-017-0410-8 PMC5639767

[27] Jaumdally H , Kwok M , Jama Z , et al. Basophil activation test has high reproducibility and is feasible in the clinical setting. Pediatr Allergy Immunol. 2022;33(11):e13870. 36433860 10.1111/pai.13870 PMC9828203

[28] Keswani T , Patil SU . Basophil activation test in food allergy: is it ready for real‐time? Curr Opin Allergy Clin Immunol. 2021;21(5):442‐447. 34374669 10.1097/ACI.0000000000000774 PMC8629129

