# Supplementary material for: Basophil allergen threshold sensitivity to casein (casein‐specific CD‐sens) predicts allergic reactions at a milk challenge in most but not all patients
Source: Immun Inflamm Dis. 2024 May 9;12(5):e1265. doi: 10.1002/iid3.1265 (PMC11080960; doi:10.1002/iid3.1265)
Supplement: Supplementary file 1 — Supporting information. [file IID3-12-e1265-s001.pdf]

E-figure 1

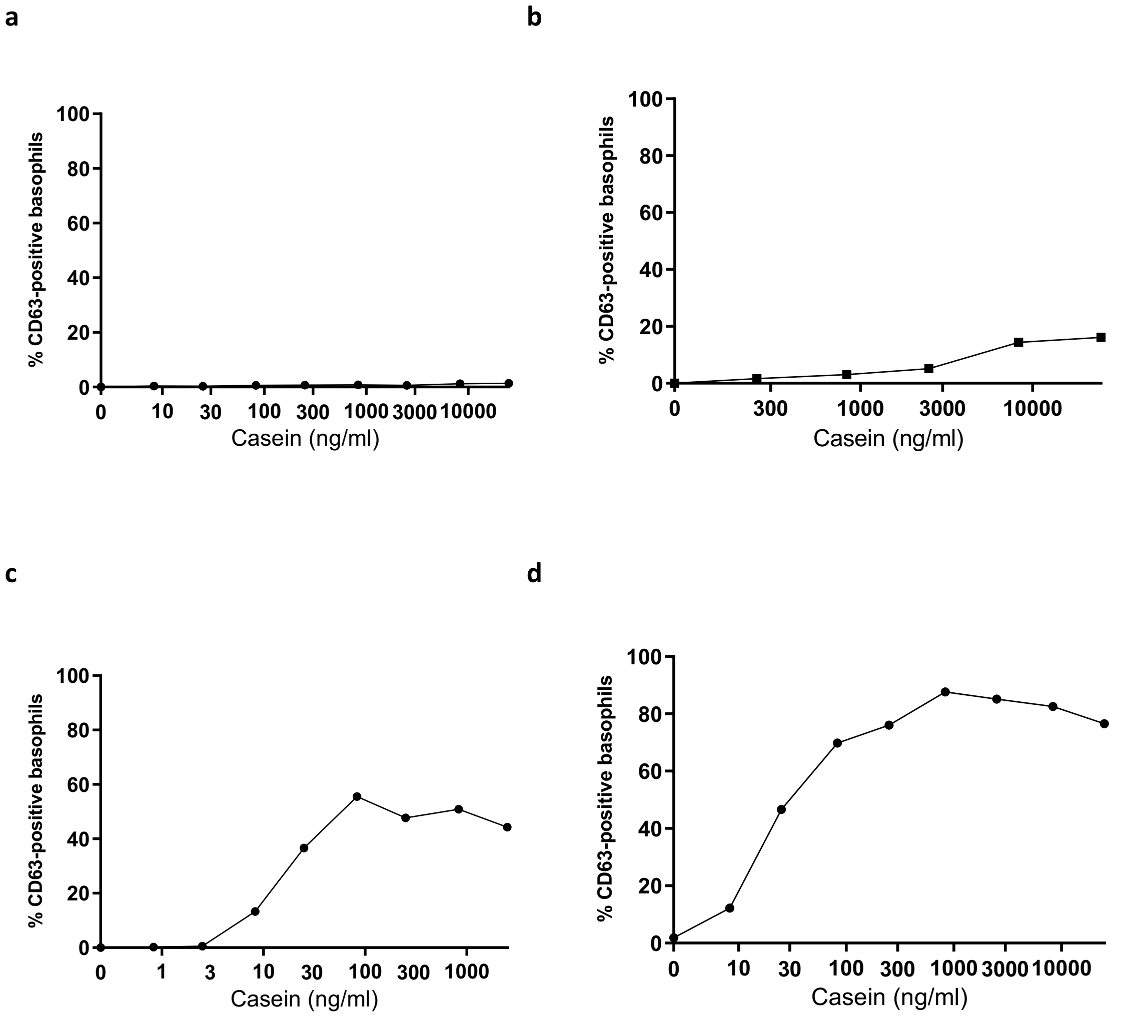

E-Figure 1. Representative dose-response curves from negative to positive reactions.

E-figure 2

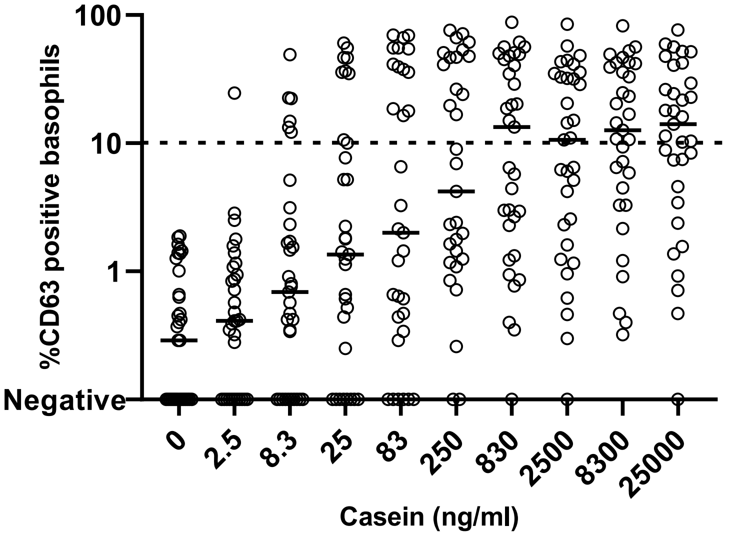

E-Figure 2. The individual responses from all patients and all casein concentrations are shown. The median response to each concentration is presented with a line and the cut off for a positive test i.e. 10% is presented with a dashed line.
